# Effects of Woodsmoke Exposure on Airway Inflammation in Rural Guatemalan Women

**DOI:** 10.1371/journal.pone.0088455

**Published:** 2014-03-13

**Authors:** Michael J. Guarnieri, Janet V. Diaz, Chandreyi Basu, Anaite Diaz, Daniel Pope, Kirk R. Smith, Tone Smith-Sivertsen, Nigel Bruce, Colin Solomon, John McCracken, John R. Balmes

**Affiliations:** 1 Department of Medicine, University of California San Francisco, San Francisco, California, United States of America; 2 Medical Entomology Research, University del Valle, Guatemala City, Guatemala; 3 Department of Public Health and Policy, University of Liverpool, Liverpool, United Kingdom; 4 Environmental Health Sciences, School of Public Health, University of California, Berkeley, California, United States of America; 5 Research Unit for General Practice, Uni Research, Bergen, Norway; 6 School of Health and Sport Sciences, University of the Sunshine Coast, Queensland, Australia; 7 Emerging Infectious Disease Unit, University del Valle, Guatemala City, Guatemala; The Ohio State University, United States of America

## Abstract

**Background:**

More than two-fifths of the world’s population uses solid fuels, mostly biomass, for cooking. The resulting biomass smoke exposure is a major cause of chronic obstructive pulmonary disease (COPD) among women in developing countries.

**Objective:**

To assess whether lower woodsmoke exposure from use of a stove with a chimney, compared to open fires, is associated with lower markers of airway inflammation in young women.

**Design:**

We carried out a cross-sectional analysis on a sub-cohort of participants enrolled in a randomized controlled trial in rural Guatemala, RESPIRE.

**Participants:**

We recruited 45 indigenous women at the end of the 18-month trial; 19 women who had been using the chimney stove for 18–24 months and 26 women still using open fires.

**Measurements:**

We obtained spirometry and induced sputum for cell counts, gene expression of IL-8, TNF-α, MMP-9 and 12, and protein concentrations of IL-8, myeloperoxidase and fibronectin. Exhaled carbon monoxide (CO) and 48-hr personal CO tubes were measured to assess smoke exposure.

**Results:**

MMP-9 gene expression was significantly lower in women using chimney stoves. Higher exhaled CO concentrations were significantly associated with higher gene expression of IL-8, TNF-α, and MMP-9. Higher 48-hr personal CO concentrations were associated with higher gene expression of IL-8, TNF- α, MMP-9 and MMP-12; reaching statistical significance for MMP-9 and MMP-12.

**Conclusions:**

Compared to using an open wood fire for cooking, use of a chimney stove was associated with lower gene expression of MMP-9, a potential mediator of airway remodeling. Among all participants, indoor biomass smoke exposure was associated with higher gene expression of multiple mediators of airway inflammation and remodeling; these mechanisms may explain some of the observed association between prolonged biomass smoke exposure and COPD.

## Introduction

Household air pollution from cooking with solid fuel (wood, dung, crop residues, coal, charcoal) is estimated to be responsible for 4.6% of worldwide disability adjusted life-years lost. [1] This is due to acute lower respiratory infections (ALRI) in children, chronic obstructive pulmonary disease (COPD), lung cancer, cataracts, and cardiovascular disease in adults. [2] More than two-fifths of the world’s population uses solid fuel as their primary fuel for cooking and the vast majority of these people do so over open fires indoors. [3] This inefficient way of cooking exposes women (who tend to perform the majority of cooking) on a daily basis to high levels of smoke. Mean indoor 24-hour concentrations of fine particulate matter (particles with aerodynamic diameter of <2.5 microns, PM_2.5_), a component of biomass smoke, have been found to be up to 100 times higher than the air quality guidelines of the World Health Organization (10 ug/m3 annual average) and are commonly 30 times higher [4], [5].

Epidemiologic studies of women from developing countries have shown strong associations between use of biomass cooking fuel and chronic bronchitis and/or COPD. [6]–[15] Separate meta-analyses by Hu, et al. and Kurmi, et al identified an increased risk of COPD among those exposed to biomass smoke with odds ratios of 2.44 (95% CI, 1.9–3.33) and 2.80 (95% CI, 1.85–4.0), respectively. [16], [17] A prospective study showed that participants with COPD due to biomass smoke have similar mortality rates as participants with COPD due to tobacco smoke [18].

Multiple studies have identified an increase in inflammatory mediators in sputum that may suggest a mechanism for this increased risk. One study of COPD due to biomass smoke exposure sampled respiratory tract lining fluid by bronchoalveolar lavage (BAL), finding increased matrix metalloproteinase (MMP)-12 gene expression and activity and increased MMP-9 activity. [19] More recently, observational studies of individuals in West Bengal, India exposed to a combination of solid fuel types (wood, dung, and crop residues) found an increase in inflammatory cells and other markers of inflammation in expectorated sputum, including total cell counts, leukocyte counts, and sputum levels of interleukin (IL)-6, IL-8, tumor necrosis factor (TNF)-α, and reactive oxygen species after adjustment for confounders, though this study was limited by significant differences between the biomass smoke-exposed and comparison (liquid petroleum gas stove) groups at baseline. [20]–[22] Our study has several advantages over previous literature on this topic, as subjects were recruited following a randomized stove intervention trial and more intensive exposure monitoring was collected on participants including exhaled breath CO and 48-hour personal CO concentrations.

We carried out our study in rural highland Guatemala, the site of a randomized controlled trial (Randomised Exposure Study of Pollution Indoors and Respiratory Effects - RESPIRE) in which households using open fires indoors for cooking were randomly assigned to receive a well-operating chimney stove (locally called the Plancha), either at the start or end of the study, after an 18-month study period. [23] The main outcomes of RESPIRE were incidence of acute lower respiratory infections in infants and respiratory symptoms and lung function in their mothers. Measurements showed that the chimney stoves, which were indigenously developed in Guatemala and have been deployed widely since the late 1980s, were popular, sturdy, and reduced kitchen air pollution and personal exposures to women and children [24], [25].

We used induced sputum to obtain samples of respiratory tract lining fluid from a subgroup of young women enrolled in RESPIRE at the end of the 18-month trial, to determine if reduced smoke exposure from use of the chimney stove is associated with decreases in markers of airway inflammation and injury that characterize COPD. [26]–[30] We chose to examine biomarkers suggestive of macrophage and epithelial cell activation (IL-8, TNF-α, MMP-9 and 12), neutrophilic inflammation (differential cell counts, myeloperoxidase and MMP-9), and injury (fibronectin), each of which have been linked to COPD pathogenesis in previous studies.

## Methods

### Ethics Review

The study protocol was approved by the institutional review boards at the University of California, San Francisco; the Universidad del Valle, Guatemala; the University of Bergen, Norway; and the University of Liverpool, United Kingdom. Because of the low literacy rate in this population, oral informed consent was obtained in each participant’s native language and documented by field workers using a standardized form. This method of consent was approved by each institutional review board.

### Study Design and Participants

RESPIRE was a randomized controlled stove intervention trial undertaken between October 2002 and December 2004 in which households with children up to 18 mo of age were randomized either to receive a stove with a chimney or to continue cooking over an open fire until the end of the trial. Details of the RESPIRE protocol have been published elsewhere. [31], [32] Participants of this cross-sectional study were all mothers of children participating in RESPIRE, recruited in December 2004 near the end of the 18-month study period. A convenience sample of 50 women was invited to participate in this cross-sectional study and 45 accepted, with 26 from open-fire and 19 from chimney-stove homes. All tests were done at the Ministry of Health clinic in San Lorenzo over a 2-week period, where participants underwent spirometry and sputum induction to assess for airway markers of inflammation and injury.

Women were assessed by self-report for the following exclusion criteria: a) acute respiratory infection within the past 4 weeks; b) use of steroids; c) history of current or former smoking; or d) lack of participation in cooking. One (2%) woman was excluded because of acute respiratory infection.

### Spirometry

Using a portable spirometer (EasyOne, ndd), we measured forced expiratory volume in 1 second (FEV_1_) and forced vital capacity (FVC) before and 15 minutes after administration of a bronchodilator. [33] Only measurements that were acceptable and reproducible by 1994 American Thoracic Society criteria were analyzed. [34], [35] Reversibility was defined as ≥12% and ≥200 ml increase in FEV_1_ or FVC, and COPD as post-bronchodilator FEV_1_/FVC ratio <0.70 [36]. Reference values were calculated from the NHANES III-derived equation for Mexican-Americans. [34] Concavity of expiratory flow-volume curves was assessed by a pulmonologist blinded to the RESPIRE stove intervention status.

### Sputum Induction and Processing

One of the investigators induced and processed the sputum and performed total cell counts at the Ministry of Health clinic with the assistance of a RESPIRE field worker. The cell slides and frozen sputum samples (supernatant and cells) were transported to the Lung Biology Center at the University of California, San Francisco.

### Cell Counts, Gene Expression and Protein Concentrations

Differential cell counts (400 non-squamous cells) were performed in duplicate by two independent counters who were blinded to the RESPIRE stove intervention status. All gene expression and protein assays were conducted at the Lung Biology Center by one of the investigators. Concentrations of myeloperoxidase (MPO), IL-8 and fibronectin in sputum supernatants were measured using commercially available ELISAs (MPO: Calbiochem, Germany; IL-8: R&D Systems, Minneapolis, USA; fibronectin: Biomedical Technologies, Massachusetts, USA). Relative quantification of IL-8, TNF-α, MMP-9 and MMP-12 gene expression was performed by comparison to an endogenous control, glyceraldehyde 3-phosphate dehydrogenase (GAPDH), using the two-step Real-Time Quantitative Reverse Transcription-Polymerase Chain Reaction (Applied Biosystems, TaqMan) according to standard protocols available at http://docs.appliedbiosystems.com/pebiodocs/04371095.pdf).

### Personal 48-hr Carbon Monoxide (CO)

Personal 48-hr CO concentrations [reported in parts per million (ppm)] were measured using Dräger Safety (Luebeck, Germany) passive diffusion tubes, which were carried by participants in small loosely woven bags previously shown not to impede diffusion of CO. [24] The passive diffusion tubes function by changing colors as CO passes through the tubes, with the length of the color stain being proportional to total CO exposure in ppm over the 48-hour period. Exhaled breath CO and personal 48-hr CO monitoring were used in this study as surrogates for biomass smoke exposure; Personal CO been shown to be a reliable surrogate for PM_2.5_ exposure due to biomass combustion. [37] Quality control and calibration of personal CO measurements were maintained according to previously described protocols. [4] These were collected at least two times after stove installation (generally 0, 3, 6, and 12 months). As the number of personal CO measurements and timing relative to sputum induction was variable, the last 48-hr personal CO measurements obtained prior to sputum induction were used for regression analyses.

### Exhaled Carbon Monoxide Measurement

During the 18-month duration of the RESPIRE study, exhaled CO concentration (in ppm) was measured for each participant using the Micro CO Meter (Micro Medical) at three separate times (6, 12 and 18 months). Exhaled CO measurements were not obtained during the induced sputum visits. The maneuver required the participant to hold her breath for 15 seconds and exhale completely with her lips sealed around a disposable mouthpiece. At each testing session, the participant performed the maneuver three times and the two highest readings were used in analysis. The mean value of the six highest readings obtained during the three testing sessions was used as the predictor variable in exposure-responses analyses. Two women were unable to complete any exhaled CO measurements. Four women completed two of three exhaled CO measurements; their mean CO was calculated as the mean of the two highest reading taken at each measurement. Exhaled CO monitors were calibrated against a standard CO span gas every 2–4 weeks during the study period.

### Statistical Analysis

Statistical analysis was performed using Stata software version 12.1 and SAS software version 9.1.3. A sample size of 17 per group was calculated to detect a difference of 10% in percent neutrophils between the groups, with type 1 error of 0.05 (two-sided) and power of 80%. Univariate comparisons between the two groups were performed using unpaired student t-Tests for normally distributed continuous variables or their logarithmic transformations, Wilcoxon rank sum for non-normally distributed continuous variables, and chi-square or Fisher exact tests for categorical variables. Normally distributed data are expressed as mean values with standard deviation (SD) and non-normally distributed data are expressed as median values with interquartile ranges (IQR).

Simple linear regression models were used to describe exposure-outcome associations, with untransformed exhaled CO concentration (ppm) and 48-hr personal CO concentrations as the independent variable and the inflammatory markers or their logarithmic transformations as dependent variables; Simple linear regression was also used to describe the relationship between lung function and percent sputum neutrophils. Distributions were graphically assessed and variables were log-transformed for regression analysis when non-normally distributed (resulting in log transformation of all gene products). Assumptions for linear regression were assessed using standardized residual versus fitted value and normal quantile plots (to assess functional form and constant variance assumptions) and Shapiro Wilk tests were used to formally assess for normality of residual distributions; assumptions were met for all included regression analyses.

## Results

### Participant Characteristics

The characteristics of women, at the time of induced sputum visits, from open-fire and chimney-stove homes are shown in Tables 1 and 2. There were no significant differences in age, height, weight, village, highest educational attainment (for participant or spouse), primary cooking location, or in the following pre-or post-bronchodilator spirometric indices: percent predicted FEV_1_, percent predicted FVC, and FEV_1_/FVC ratio. Although the percentages of women with no formal education and with their primary cooking area in the same room as the bedroom were higher in the open fire group, these differences were not significant. Thirty-five (78%) women had acceptable (as determined by American Thoracic Society performance criteria) pre- and post-bronchodilator spirometric measurements, an additional six (13%) women had acceptable pre- or post-bronchodilator spirometric measurements, and four (9%) women had unacceptable pre- and post- measurements. Most (16 out of 19 women, or 84%) had the chimney stove for at least 18 months, with the remainder having their stove at least 15 months. The participants from the open-fire group did have more cases of both concavity of expiratory flow volume curve (50% versus 30%, p = 0.22) and airflow reversibility (16% versus 0%, p = 0.23), although these differences were not statistically significant. Though no participant was known to have respiratory disease at the time of enrollment, one participant was identified as having COPD based on spirometry performed during the study.

**Table 1:** Characteristics of study participants.

Characterisitics	Chimney stove		Open fire				
	n = 19		n = 26				
	mean	sd	mean	sd	Mean difference	95%CI	p-value
***Age*** (years)	26.6	7.3	26.4	5	0.2	3.9, −3.5	0.89
***Height*** (cms)	144.6	4.1	145.4	4.3	−0.8	−3.3, 1.8	0.56
***Weight*** (kgs)	49.7	6.7	50.8	8	−1.1	−5.7, 3.4	0.63
***Exhaled CO*** (ppm)	5.2	1.1	7.1	1.6	−1.9	−1.1, −2.8	<0.001
***Personal CO tube*** (ppm)	1.5	1.2	3.5	1.7	−2	−1.0, −2.9	<0.001
***Cooking location:***	**no.**	**%**	**no.**	**%**			**p-value**
Main house (separated by a wall)	1	5.3	1	3.9			0.67
Main house (same area as bedroom)	1	5.3	4	15.4			
Separate enclosed cooking structure	15	79	17	65.4			
Separate open structure (1+ wall missing)	2	10.5	4	15.4			
***Community:***							
Tiuxoquel	6	31.6	9	34.6			0.65
Tuichilupe	11	57.9	12	46.2			
Vista Hermosa	2	10.5	5	19.2			
***Highest education (participant):***							
None	5	26.3	11	42.3			0.31
Primary school	13	68.4	15	57.7			
Secondary school	1	5.3	0	0			
***Highest education (spouse):***							
None	2	10.5	5	19.2			0.77
Primary school	11	57.9	15	53.9			
Secondary school	2	10.5	4	15.4			
***Occupation:***							
Agricultural work	13	68.4	17	65.4			0.83
Non-agricultural work	6	31.6	9	34.6			

**Table 2:** Lung function of study participants.

Pre-bronchodilator spirometric indices	Chimney stove n = 17	Open fire n = 20	p-value	95%CI for difference
FEV1, percent predicted (mean, SD)	102 (13)	103 (12)	0.76	−7, 10
FVC, percent predicted (mean, SD)	108 (13)	111 (13)	0.4	−5, 12
FEV1/FVC % (mean, SD)	0.82 (0.07)	0.80 (0.05)	0.54	−0.06, 0.03
*Concavity, n (%)	5 (30)	10(50)	0.22	
**Post-bronchodilator spirometric diagnostics**	**n = 16**	**n = 19**		
*Reversibility, n (%)	0	3 (16)	0.23	
*COPD, n (%)	1(6)	0	0.46	
**Pre-bronchodilator spirometric indices**	**Chimney stove n = 17**	**Open fire n = 20**	**p-value**	**95%CI for difference**
FEV1, percent predicted (mean, SD)	102 (13)	103 (12)	0.76	−7, 10
FVC, percent predicted (mean, SD)	108 (13)	111 (13)	0.4	−5, 12
FEV1/FVC % (mean, SD)	0.82 (0.07)	0.80 (0.05)	0.54	−0.06, 0.03
*Concavity, n (%)	5 (30)	10(50)	0.22	
**Post-bronchodilator spirometric diagnostics**	**n = 16**	**n = 19**		
*Reversibility, n (%)	0	3 (16)	0.23	
*COPD, n (%)	1(6)	0	0.46	

*Data are categorical variables and the percent represents number of participants with concavity or reversibility divided by total number of participants in each group.

### Exposure Characteristics

Exhaled CO measurements were available for 43 women and 48-hr personal CO measurements were available for all participants. Participants from the open-fire homes (n = 26) had significantly higher mean exhaled CO concentrations than women from chimney-stove homes (n = 17), with 7.1 ppm (SD 1.6) and 5.2 ppm (SD 1.1), respectively, with a mean difference of 1.96 ppm (95% CI 1.1–2.8, p = 0.0003). Women from open fire homes (n = 26) also had significantly higher 48-hr personal CO concentrations than women from chimney-stove homes (n = 19), 3.5 ppm (SD 1.7) and 1.5 ppm (SD 1.2), respectively, with a mean difference of 2.0 ppm (95% CI 1.0–2.9, p<0.001). Pearson’s correlation coefficient for exhaled CO and 48-hr personal CO was 0.48.

### Induced Sputum Quality and Cell Counts

**Figure 1:**
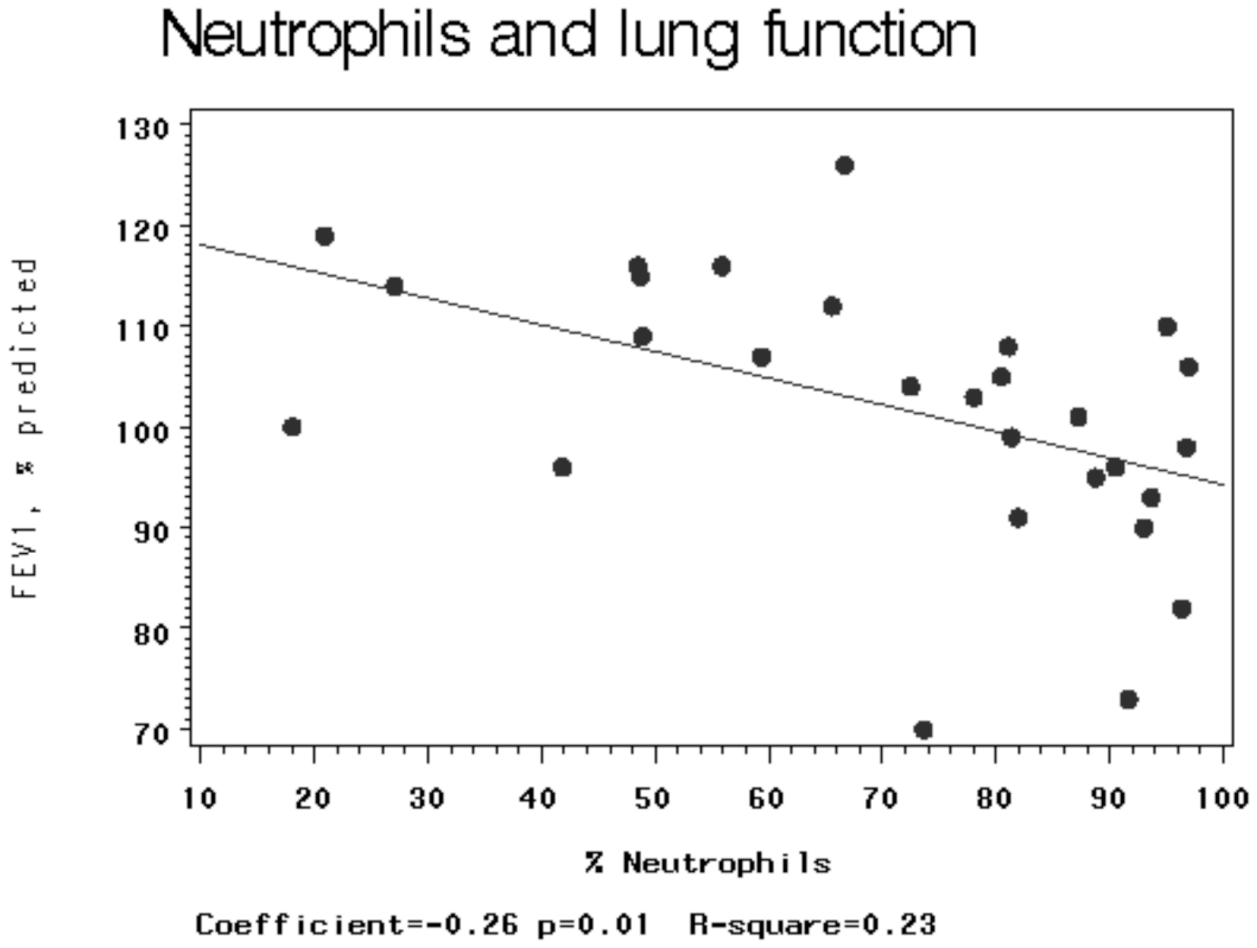
Simple linear regression between percent neutrophils (independent variable) and percent predicted FEV_1_ (dependent variable). Dots represent individual estimates. Solid line is made to linear best fit model.

Of the 45 women eligible for sputum induction, 40 (89%) women produced sputum and 31 (69%) women had cell slides of acceptable quality for use in the analysis: 14 from open-fire and 17 from chimney-stove homes. Total and differential cell counts are shown in Table 3. There were no significant differences between the groups in total leukocytes, differential counts of neutrophils, macrophages, eosinophils and lymphocytes. Percent epithelial cells was significantly higher in participants from the open-fire group in comparison to the chimney-stove group (p = 0.05). In post hoc analysis, percent neutrophils in sputum was negatively associated with percent predicted FEV_1_ (β = −0.26, p = 0.01; Figure 1).

**Table 3:** Induced sputum cell counts of study participants.

Cell concentrations	Chimney stove	Open fire	p-value
	(n = 14)	(n = 17)	
Total leukocytes × 10^4^/ml (mean, SD)	234.4 (171)	255.2 (172)	0.7
Neutrophils × 10^4^/ml (mean, SD)	187.8 (167)	189.6 (174)	0.71
Macrophages × 10^4^/ml (mean, SD)	54.9 (52)	88.7 (94)	0.61
**Differential cell counts**			
†Neutrophils, % WBC (median, IQR)	79.3 (59.4, 87.3)	72.5 (48.5, 90.6)	0.58
†Macrophages, % WBC (median, IQR)	20.7 (12.7, 40.1)	27.5 (9.2, 51.5)	0.63
‡Epithelial cells, % all (median, IQR)	1.25 (0.30, 2.0)	2.0 (1.3, 3.3)	0.05

† The mean of cell type divided by the sum of all white blood cells multiplied by 100.

‡ The mean of epithelial cells divided by sum of all cells multiplied by 100.

### Induced Sputum Protein Concentrations

Of the 40 women who produced sputum, 32 (80%) women produced enough sputum for protein analysis. Of these, 22 (69%) women had samples that were sufficient in quantity to carry out all three of the protein assays. Mean protein concentrations of IL-8 were lower in the chimney-stove group (1801 ng/ml versus 2573 ng/ml, p = 0.15) while mean fibronectin (485 ng/ml versus 355 ng/ml, p = 0.44) and median MPO (78 ng/ml versus 26 ng/ml, p = 0.40) concentrations were higher in this group, though none of these differences reached statistical significance (Table 4).

**Table 4:** Induced sputum protein concentrations of study participants.

Protein concentration	Chimney stove	Open fire	p-value
IL-8, ng/ml (mean, SD) *(n = 32)*	1801 (1406) *(n = 15)*	2573 (1533) *(n = 17)*	0.15
Fibronectin, ng/ml (mean, SD) *(n = 22)*	485 (409) *(n = 11)*	355 (374) *(n = 11)*	0.44
Myeloperoxidase, ng/ml (median, IQR) *(n = 29)*	78 (16, 625) *(n = 15)*	26 (14, 463) *(n = 14)*	0.4
**Protein concentration**	**Chimney stove**	**Open fire**	**p-value**
IL-8, ng/ml (mean, SD) *(n = 32)*	1801 (1406) *(n = 15)*	2573 (1533) *(n = 17)*	0.15
Fibronectin, ng/ml (mean, SD) *(n = 22)*	485 (409) *(n = 11)*	355 (374) *(n = 11)*	0.44
Myeloperoxidase, ng/ml (median, IQR) *(n = 29)*	78 (16, 625) *(n = 15)*	26 (14, 463) *(n = 14)*	0.4

### Induced Sputum Gene Expression

Good quality mRNA is difficult to extract from induced sputum because samples tend to have few cells and these difficulties may be enhanced when initial processing is carried out at a rural field station. [38] Despite these challenges, we recovered RNA from the 34 samples available for gene expression analysis and 29 samples (85%) were of good quality. Three (9%) assays were rejected because duplicate C_t_ measurements differed by more than 0.5. MMP-12 and -9 gene expression was low in the samples - all samples had mean C_t_ levels above 28. As the accuracy of gene expression measurements at such low concentrations can be limited, we carried out our analyses both including and excluding samples with very low levels of gene expression (defined as mean C_t_ levels >37) and found similar results (n = 22 for MMP-9, n = 27 for MMP-12). We used the Comparative C_t_ Method to quantify our gene of interest as the number of gene copies per 10^4^ copies of GAPDH because validation experiments revealed efficiencies of GAPDH, IL-8, TNF-alpha and MMP-9 to be above 90%. The chimney-stove group had significantly less MMP-9 gene expression than the open-fire group (0.002 vs 0.01, p = 0.01). In addition, gene expression of TNF-alpha was more than 50% lower and gene expression of MMP-12 was 10 fold lower in the chimney stove group, though these differences were not significant (Table 5).

**Table 5:** Induced sputum gene expression of study participants.

Gene copies per 10^4^ copies of GAPDH†	Chimney stove	Open fire	p-value
TNF-α (median, IQR)	299 (111, 509)	682 (165, 1566)	0.12
(n = 29)	*(n = 14)*	*(n = 15)*	
IL-8 (median, IQR)	519 (20, 2016)	575 (268, 1349)	0.55
(n = 29)	*(n = 14)*	*(n = 15)*	
MMP-12 (median, IQR)	0.01 (0.005, 0.08)	0.10 (0.02, 0.22)	0.17
(n = 27)	*(n = 14)*	*(n = 13)*	
MMP-9 (median, IQR)	0.002 (0.0008, 0.006)	0.01 (0.003, 2.96)	0.01
(n = 22)	*(n = 12)*	*(n = 10)*	

† glyceraldehyde 3-phosphate dehydrogenase.

### Exposure-Outcome Associations

Exhaled CO concentrations (in ppm) were positively associated with MMP-9 (ß = 2.17, 95% CI 1.2–3.9), TNF-α (ß = 1.3, 95% CI 1.0–1.7) and IL-8 (ß = 1.64, 95% CI 1.1–2.4) gene expression in log copies per 10^4^ copies of GAPDH (Table 6). Personal 48-hr CO concentrations (in ppm) were positively associated with MMP-9 (ß = 2.58, 95% CI 1.5–4.4) gene expression. Additionally, there were positive, non-significant associations between 48-hr personal CO exposure with MMP-12 (ß = 0.4, 95%CI 0.96–2.0), IL-8 (ß = 1.41, 95% CI 0.98–2.0) and TNF-α (ß = 1.22, 95% CI 0.98–1.5) gene expression. There were no significant relationships between concentrations of either exhaled CO or personal 48-hr CO exposure and fibronectin, IL-8, MPO protein concentrations, percent neutrophils, or continuous spirometric variables (% predicted FEV1, % predicted FVC, or FEV1/FVC ratio).

**Table 6:** Exposure-outcome relationships between exhaled concentration of CO and inflammatory markers.

Gene copies per 10^4^	Exhaled Breath CO (per 1 ppm)		Personal CO Tube (per 1 ppm)	
copies of GAPDH†	Coefficient	95% CI (p-value)	Coefficient	95% CI (p-value)
MMP-9 (n = 22)	2.17‡	1.2, 3.9 (0.01)	2.58‡	1.5, 4.4 (0.01)
TNF-α (n = 29)	1.30‡	1.0, 1.7 (0.04)	1.22‡	0.98, 1.5 (0.08)
IL- 8 (n = 29)	1.64‡	1.1, 2.4 (0.02)	1.41‡	0.98, 2.0 (0.06)
MMP-12 (n = 27)	0.93‡	0.6, 1.3 (0.84)	1.49‡	1.0, 2.2 (0.05)
**Protein concentrations ng/ml**				
Fibronectin	−8.6	−128, 111 (0.88)	−63.5	−152, 25.3 (0.15)
Myeloperoxidase	4.3	−88.6, 97.3 (0.92)	−26.9	−104, 50.7 (0.48)
IL-8	170	−134, 474 (0.26)	49.3	−245, 343 (0.73)
**Differential cell counts**				
Neutrophils, %WBC	0.2	−5.1, 5.5 (0.93)	1.11	−4.5, 6.6 (0.68)

† glyceraldehyde 3-phosphate dehydrogenase.

‡ Log-linear scale after back-transformation.

## Discussion

We took advantage of a unique opportunity – a randomized stove intervention trial – to compare inflammatory markers in sputum between differentially woodsmoke exposed, young, indigenous Guatemalan women. Our cross-sectional analysis shows that women from the chimney-stove homes had significantly fewer epithelial cells and lower MMP-9 gene expression in induced sputum when compared to women from open-fire homes. Exposure-outcome analyses demonstrated significantly higher levels of IL-8, TNF-α, MMP-12 and MMP-9 gene expression associated with higher levels of biomass smoke exposure, using personal CO and exhaled breath CO as surrogates for exposure. Additionally, the direction of the association between all examined gene expression products and these surrogates for biomass smoke exposure was positive, with higher expression of all gene products at higher levels of biomass exposure, with the exception of MMP12 and exhaled CO. As the protein products of these genes are associated with COPD pathogenesis in tobacco smokers, these findings suggest that important commonalities may exist between tobacco and biomass smoke-associated COPD pathogenesis, particularly if these findings are replicated and extended in larger longitudinal studies.

Those with COPD due to tobacco smoke have been shown to have increased levels and activity of matrix metalloproteases (MMP-8, -9, and 12) as well as IL-8 and TNF-α in induced sputum and BAL fluid. [39]–[47] IL-8 and TNF-α are potent cytokines released by activated epithelial cells and macrophages. MMPs are proteases that are involved in tissue growth, injury and repair as well as modulating inflammatory cascades. [48] To our knowledge, only one previous study has examined MMP levels in women with COPD due to biomass smoke and found increased levels of MMP-9 activity and increased MMP–12 gene expression and activity in BAL when compared to healthy controls. [19] Although sputum induction samples both proximal and distal airway lining fluid, our data are consistent with and extend these findings. While most of our participants had normal spirometry, the prevalence of mild airflow obstruction (concave expiratory flow-volume curves) in these young women does suggest the potential for the development of woodsmoke-induced COPD. Given the observed associations between smoke exposure and gene expression identified in this study, reductions in smoke exposure with a cleaner stove and/or fuel type may prevent the chronic pulmonary inflammation and airway remodeling that culminates in COPD in many such biomass smoke exposed women.

This study has several noteworthy limitations. Given the cross-sectional nature, we cannot say with certainty that the observed differences in gene expression are due to the chimney stove, as baseline levels for these biomarkers were not collected and the identified abnormalities could have preceded the intervention. There is some risk of selection bias since we employed convenience sampling; while participants were largely selected based on proximity to the field office, it is possible that participants with a larger reduction in indoor smoke, symptoms, or both may have chosen to participate. This sampling strategy also limits the generalizability of these results as this sample may not adequately represent biomass smoke-exposed women both inside and outside of Guatemala. As exposures were not collected immediately prior to outcome measures and only represent short periods of exposure, there is a risk of non-differential exposure misclassification, which most frequently results in an underestimate of the true effect size. Additionally, while gene expression was elevated for multiple genes implicated in COPD pathogenesis, the protein products or activity of several of these genes were either not measured (for MMP-9, MMP-12, and TNF-α) or were not convincingly associated with biomass smoke exposure (for IL-8). Concordant results for gene expression and protein product analyses would strengthen the observations in this study. Finally, our assessment of multiple outcomes increases the risk of a Type I error, though the consistency of the findings serves to diminish this concern to some degree.

Unlike recent observational studies in West Bengal, which reported lower numbers of airway neutrophils among individuals using LPG stoves compared to biomass stoves, we did not identify a similar finding in the chimney-stove group in our study. [20], [22] The persistence of high airway neutrophils in the group receiving a chimney stove may be explained by the long historical exposure to woodsmoke of our participants, rather than their more recent exposure. Assuming relatively stable time-activity patterns in this young, homogenous group of women, we examined the association between age and sputum percent neutrophils. Although the mean age of our participants was only 26 years, our analysis did show a trend towards an association between age and percent neutrophils (β = 1.4 p = 0.07. Data not shown). If cumulative exposure determines neutrophilic inflammation, it may take more time (i.e., more than the 18-month time period of RESPIRE) with reduced exposure to see a reduction in airway neutrophils.

Alternatively, as there was a notable overlap in exposure profiles in the chimney-stove and control groups despite significant reductions in mean personal exposures, it is possible that a threshold effect exists for airway inflammation due to biomass smoke that was exceeded by the majority of individuals in both groups. We know that women from the chimney-stove group continue to have woodsmoke PM exposure much higher than recommended by WHO Air Quality Guidelines. Collectively, these findings could have important implications for future stove intervention trials focused on reducing COPD incidence or progression due to biomass smoke exposure, suggesting that longer periods of follow-up and cleaner stove interventions may be necessary to more clearly demonstrate an effect.

Exhaled CO measurements have been used repeatedly to distinguish between smokers and non-smokers (cut-offs between 6–8 ppm) and recently, in a study from Taiwan, Hung et al. showed an association between increasing tobacco consumption and exhaled CO concentration. [49] We used exhaled CO to assess personal exposure to woodsmoke because for cultural and economic reasons tobacco smoking is rare in both men and women in our study area. We used average values from three time points as a measure of chronic exposure, although the value from each time point is reflective of acute exposure. The addition of personal 48-hour CO measurements, which should capture exposure from multiple stove burn cycles and is strongly correlated with PM_2.5_ exposure in this population, further strengthens the exposure assessment in this study. [4] Aside from being exposed to woodsmoke while cooking (two to three times a day), these women are also exposed to very high amounts of smoke during weekly bathing in a wood-fired sauna (*temazcal*). [50] Thus, the benefit of smoke reduction from using the chimney stove may be offset by the continued, although intermittent, use of the *temazcal* and this may explain why the distributions of exposure for the two groups overlap. Better metrics are necessary to more accurately characterize chronic woodsmoke exposure in future studies.

## Conclusions

Our cross-sectional analysis of rural Guatemalan women who cook indoors and were enrolled in a randomized stove intervention trial showed significantly lower MMP-9 gene expression in induced sputum cells from women who used a chimney stove in comparison to women who used traditional open fires. Among the entire cohort, higher smoke exposure was associated with higher gene expression of multiple inflammatory mediators that are implicated in the development and progression of tobacco-associated COPD, including MMP-9, MMP-12, IL-8, and TNF-α. These findings suggest that similar mechanisms may be responsible for tobacco and biomass smoke-associated COPD pathogenesis. The persistence of pulmonary inflammation and overlapping exposure profiles in the control and intervention groups suggest the need for larger, longitudinal study to examine the efficacy of cleaner stove or fuel interventions for the prevention of COPD in young women with high cumulative lifetime exposures to biomass smoke. Such a study is of utmost importance since nearly one-half of the world’s population is exposed to biomass smoke and COPD is expected to become the 5^th^ leading cause of the global burden of disease by 2012 [51].
